# The association between serum heat shock protein 72 and intestinal permeability with intestinal microbiota and clinical severity in patients with cerebral infarction

**DOI:** 10.3389/fmed.2023.1302460

**Published:** 2024-01-09

**Authors:** Jiahui Zhu, Yijie Dai, Bo Tang, Hao Zhang

**Affiliations:** Department of Neurology, Hangzhou First People’s Hospital, Hangzhou, China

**Keywords:** intestinal flora, cerebral infarction, serum heat shock protein 72, intestinal permeability, zonulin, clinical severity of CI, ELISA, NIHSS

## Abstract

**Objectives:**

We aimed to compare serum heat shock protein 72 (HSP72) and intestinal permeability in patients with cerebral infarction (CI) and healthy individuals to reveal their correlations and link to gut microbiota alterations and clinical severity of CI.

**Methods and results:**

Stool samples of 50 patients with CI and 46 healthy volunteers were analyzed through 16S rRNA gene sequencing to characterize intestinal flora profiles. Serum HSP72 and zonulin were assayed using enzyme-linked immunoassay (ELISA). The obtained data were then subjected to comparative and correlative analysis. We found that the levels of zonulin and serum HSP72 were significantly higher in the CI group compared to the healthy group. Serum HSP72 and zonulin levels were positively correlated in the CI group and correlated positively with the clinical severity of CI. β diversity showed significant differences in intestinal microbiota composition between the two groups. In the CI patient group, the abundance of bacteria *Eubacterium_fissicatena_group*, *Eubacterium_eligens_group,* and *Romboutsia* manifested a remarkably positive correlation with serum HSP72. The abundance of bacteria *Eubacterium_fissicatena_group* and *Acetivibrio* had a significantly positive correlation with zonulin levels.

**Conclusion:**

Our findings indicated that an increase in serum HSP72 and zonulin levels was manifested in patients with CI and was related to specific gut microbiota alterations and the clinical severity of CI.

## Introduction

Stroke, a devastating and lethal disease, is currently second among the leading causes of death globally and the third contributor to disability in the world (1). Besides the neurological defects caused by the infarct site, the major cause of death is the peripheral tissue damage induced by post-stroke leaky gut. Previous reports have shown that the abundance of opportunistic pathogens and the corresponding product of metabolism changes increased significantly in patients with cerebral infarction (CI) compared to healthy controls (2). Incidentally, elevated inflammatory levels after CI efficiently facilitated the increase of intestinal mucosal permeability. Strict regulation of intestinal integrity is essentially related to host antimicrobial immune defense. Regrettably, previous studies on CI and specific alterations of intestinal flora have not effectively obtained explicit conclusions.

The level of the serum zonulin, a protein produced by the intestine epithelium, is proportional to gut permeability. *In vitro* studies demonstrated that endogenous human zonulin was responsible for increased permeability in the jejunum and ileum (3). An overgrowth of intraluminal microorganism and gluten contribute significantly to intestinal zonulin release (4). Zonulin secretion has been reported to be induced through the MyD88-dependent pathway, which is followed by dissociation of the protein ZO-1 from the tight junctional complex, thus generating leaky gut syndrome (5). Of note, zonulin is the biological door to a vast number of diseases such as autoimmune diseases, neuropsychiatric diseases, and cancer through its intestinal barrier regulation function (6, 7).

Heat shock protein 72 (HSP72), a critical component of inducible HSPs (iHSPs), is a ubiquitous molecule that exerts efficient effects on cellular survivability and tolerance to stressors. Correspondingly, animal experiments showed that the upregulation of HSP72 in ischemic cerebral tissue confirmed a crucial protective role in the outcome following stroke (8). Recent evidence indicates that bacterial components and metabolites specifically control the expression of HSPs (9, 10). HSP72 can be detected in quite a few body fluids, such as pleural fluid (11), cerebrospinal (12), synovia (12), bronchoalveolar lavage fluid (13), and serum (14). Recent evidence indicated that the concentration of the stress-inducible HSP72 homolog, HSP70, increased in patients with heart failure (15) and atherosclerosis (16). Strikingly, previous studies have documented that the serum expression level of HSP70 significantly increased following acute middle cerebral artery occlusion (MCAO) in rats (16). Nevertheless, whether there is a change in human serum HSP72 after CI has not been addressed. Thereafter, how serum HSP72 correlates with intestinal dysfunction and CI severity has not been accurately explored.

Accordingly, in this study, we deciphered gut microbiome profiles through 16S rRNA gene sequencing of stool samples, quantified serum HSP72 and zonulin via enzyme-linked immunoassay (ELISA), further compared and analyzed the levels of serum HSP72 and zonulin in patients with CI and healthy controls, and explored the link between the levels of serum HSP72 and zonulin to alterations in intestinal microbiota and clinical severity of CI.

## Materials and methods

### Study subjects

This cross-sectional study was conducted at Hangzhou First People’s Hospital. We consecutively enrolled 81 patients with CI and 52 healthy individuals, respectively, from the Department of Neurology and Physical Examination Center. Herein, we rigorously screened out 50 patients with CI and 50 healthy controls who satisfied the inclusion criteria and ultimately participated in the study. Case groups were diagnosed within 2 weeks after sudden focal neurological deficits, with the acute infarct area in the corresponding brain region confirmed on the computed tomography (CT) and/or magnetic resonance imaging (MRI). Healthy controls reported never being diagnosed with any of the risk factors associated with CI, such as hypertension, diabetes, metabolic syndrome, and atrial fibrillation. Baseline characteristics such as demographic data including age, sex, dietary habits, medical histories (hypertension, diabetes, and atrial fibrillation), and laboratory data were collected. This study was approved by the Clinical Research Ethics Committee of Hangzhou First People’s Hospital.

The inclusion criteria for the study subjects were determined as (1) aged between 40 and 80 years old; (2) grew up in southern China and maintained a healthy lifestyle, with healthy dietary and bowel habits; (3) body mass index range from 18 to 24; (4) had available blood and stool samples; and (5) signed informed consent before the experiments. Subjects were excluded if they (1) had a history of intracranial hemorrhage and other neuropsychiatric disorders; (2) were previously diagnosed with respiratory failure, heart failure, uremia, severe liver dysfunction, malignant tumors, and autoimmune disease; (3) had inflammatory bowel disease, gastrointestinal bleeding, and surgery, as well as other gastrointestinal dysfunction; and (4) had received treatment with antibiotics, probiotics, or hormone drugs within 2 months before the recruitment.

### Sample collection

To avoid random error, fecal samples were taken with cotton swabs from the middle section of the stool samples provided and were kept in two 2 mL sterile frozen depository tubes with approximately 200 mg of samples in each tube. Fasting blood samples were collected in the coagulation vessels and centrifuged at 3000r for 10 min at −4°C. The serum was transferred to 1.5 mL frozen tubes. Preprocessed stool and serum samples were immediately transferred to the laboratory for storage at −80°C.

### Microbial DNA extraction and sequence data analysis

The total DNA of fecal specimens was extracted using the E.Z.N.A.^®^Stool DNA Kit and samples were sequenced on the Illumina NovaSeq platform following the manufacturer’s instructions. The complexity of the sample species diversity was characteristic of α diversity, calculated by the QIIME2. The β diversity was calculated by non-metric multidimensional scaling and plotted by the R package. Composition difference was analyzed by the Wilcoxon rank-sum test and Welch’s t-test, based on which we constructed the heat map at the phyla level.

### Quantitative assay of serum HSP72 and zonulin by ELISA

Serum HSP72 and zonulin were assayed through the Human HSP72 ELISA Kit and Zonulin ELISA Kit (Meimian Industrial, Jiangsu, China). A 50μL proof sample was added to the standard well. A 40μL sample diluent and a 10μL sample being tested were added to the enzymatic coating plate so that samples were eventually diluted to 5-fold. Samples were incubated with 100μL HRP-Conjugate reagent at 37°C for 60 min. Then, each well was filled with wash fluid, and the fluid was discarded after 30 s. This process was repeated five times, and each well was dried. Each well was incubated with chromogenic agent at 37°C for 15 min and then the reaction was terminated by adding 50μL of the stop solution to each well until the blue color turned yellow. Finally, the absorbance of each well was determined within the wavelength of 450 nm.

### Statistical analysis

Data were analyzed using SPSS version 26.0 software and R version 3.6.1 statistical software. The continuous variable was denoted by mean ± SD, while categorical variables were denoted by numbers and percentages. Measurement data meeting normality was indicated by independent sample Student’s t-test or analysis of variance and by Wilcoxon’s rank sum test if the variable violated the assumption of normality. Categorical variable differences between groups were compared by a χ^2^ test. We used binary logistic regression analysis to explore the relationship between serum HSP72 and zonulin levels and the occurrence of CI, after adjustment for age and sex. The area under the receiver operating characteristic (ROC) curve (AUC) was applied to evaluate the model’s diagnostic performance for investigating the ability of serum HSP72 and zonulin levels to distinguish between patients with CI and controls. Multivariable linear regression models were conducted to examine the association between serum HSP72 and zonulin levels and clinical severity. The correlation between intestinal flora and clinical data was assessed by Spearman’s rank correlation analysis. The data correlation conforming to the normal distribution was analyzed with the Pearson test. *p*-value <0.05 was defined as statistically significant.

## Results

### Study population and baseline characteristics

A total of 81 patients with CI and 52 healthy individuals were enrolled in this study. However, 31 patients and two control participants failed to defecate on time or provided unqualified fecal sampling, and were thus excluded from the subsequent analysis. Four healthy samples could not meet the 16S rRNA sequencing requirements due to low fecal volume, which led to unqualified amplification. Ultimately, only 96 subjects (50 patients with CI and 46 controls) remained in the next 16S rRNA analysis, and the final included samples were tested by ELISA. The National Institute of Health Stroke Scale (NIHSS) score was less than 16 in the CI group, for which the patients with severe CI had difficult fecal discharge the early next morning. All participants in the experiment were matched for age (CI group, 66.42 ± 6.92; healthy group, 65.15 ± 5.82; *p* = 0.233) and sex (M/F: case, 26/24; control, 25/21; *p* = 0.818). The demographic and baseline clinical characteristics of patients with CI and controls are shown in Table 1.

**Table 1:** Comparison of the baseline data between the two groups.

Clinical parameter	CI group (*n* = 50)	Healthy group (*n* = 46)	*p*-value
Sex, male	26 (52%)	25 (54%)	0.818
Age, y	66.42 ± 6.92	65.15 ± 5.82	0.233
NIHSS score	10.88 ± 2.67	/	/
Hypertension (*n*, %)	36 (72%)	0	< 0.01**
Diabetes (*n*, %)	25 (50%)	0	< 0.01**
Atrial fibrillation (*n*, %)	3 (6%)	0	< 0.01**
Smoking history (*n*, %)	9 (18%)	6 (13%)	0.504
TG (mmol/L)	1.53 ± 0.76	1.21 ± 0.60	0.013*
TC (mmol/L)	4.21 ± 0.97	2.64 ± 0.68	< 0.01**
LDL (mmol/L)	2.32 ± 0.84	2.43 ± 0.54	0.203
HCY (umol/L)	16.55 ± 10.90	8.81 ± 3.03	< 0.01**
UA (umol/L)	314.28 ± 89.72	350.30 ± 42.75	0.016*
Serum HSP72 (pg/g)	307.46 ± 42.59	176.61 ± 44.75	< 0.01**
Zonulin (ng/ml)	135.70 ± 30.74	105.83 ± 24.04	< 0.01**

TG, triglyceride; TC, total cholesterol; LDL, low-density lipoprotein; HCY, homocysteine; UA, uric acid; HSP72, heat shock protein 72. Variables are expressed as mean ± SD or *n* (%). ^*^p < 0.05. ^**^p < 0.01.

### Comparison of serum HSP72 and zonulin levels between the two groups

**Figure 1:**
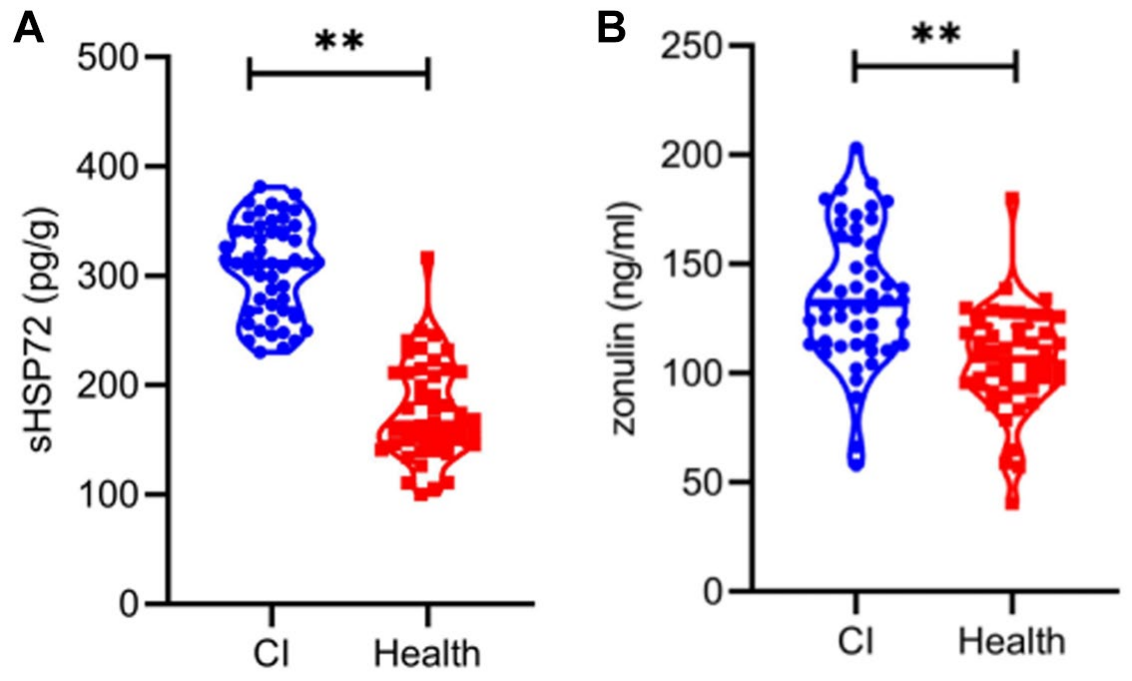
Violin plot representing data density distribution and median with interquartile range of serum HSP72 **(A)** and zonulin **(B)** between the two groups. **, *p* < 0.01.

The quantification and comparison of serum HSP72 and zonulin between the two groups are shown in Table 1 and Figure 1. The mean serum HSP72 level was 307.46 ± 42.59 pg./g in patients with CI, while the mean value was 176.61 ± 44.75 pg./g in controls. In light of this, patients with CI had significantly higher serum HSP72 than controls (*p* < 0.01). Accordingly, the mean zonulin level was 135.70 ± 30.74 ng/mL in patients with CI, while the mean value was 105.83 ± 24.04 ng/mL in controls. Moreover, serum zonulin was significantly higher in the CI group than in healthy controls (*p* < 0.01).

We then investigated whether serum HSP72 or zonulin distinguished between patients with CI and healthy individuals. Binary logistic regression analysis indicated that higher levels of serum HSP72 (OR = 1.10, 95% CI = 1.03–1.11; *p* < 0.01) and zonulin (OR = 1.04, 95% CI = 1.02–1.07; *p* < 0.01) were more likely to associated with CI patients after adjustment for sex and age.

**Figure 2:**
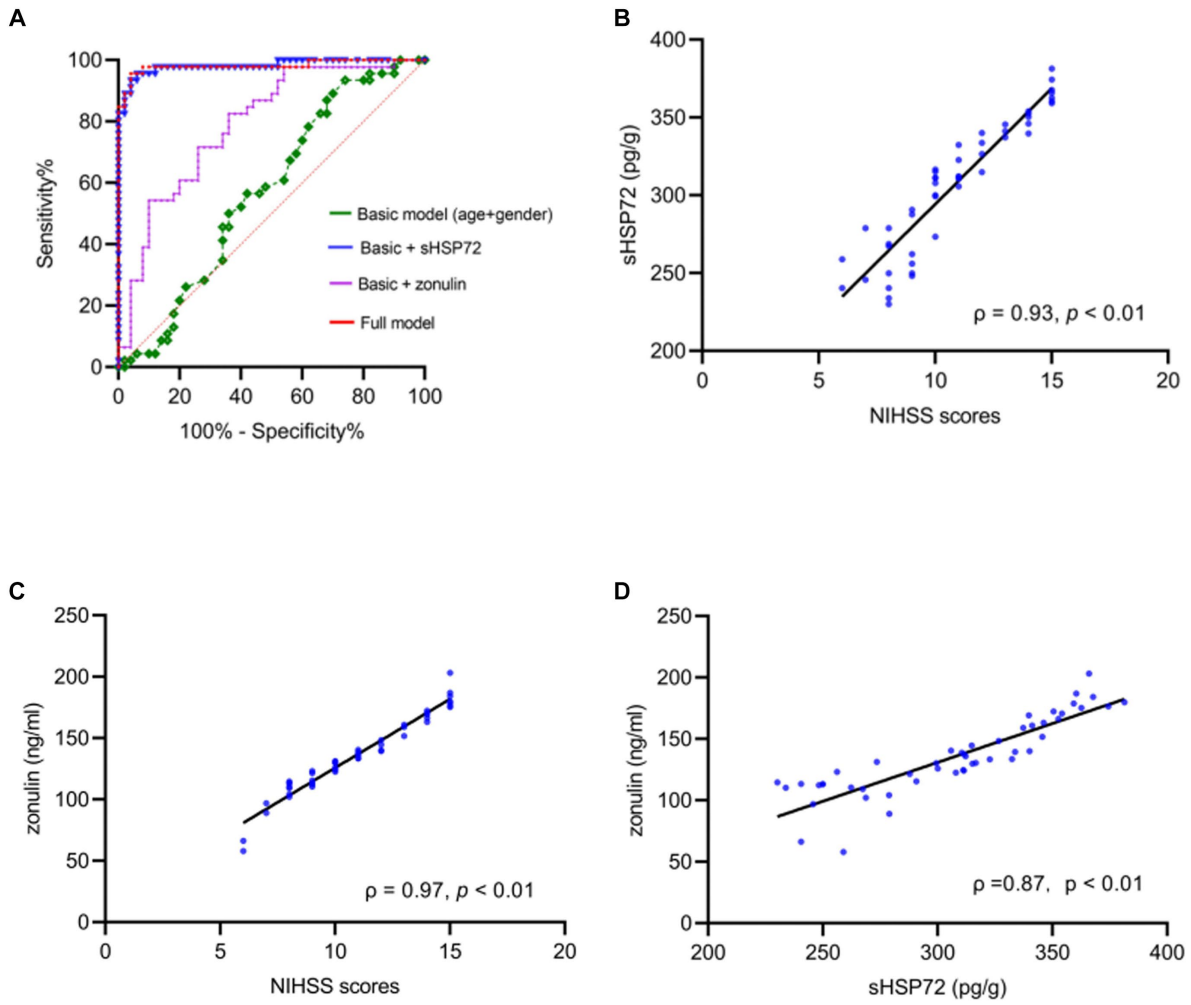
**(A)** ROC curves for estimating the accuracy of cerebral infarction (CI) using basic model (age + sex, AUC = 0.57, *p* = 0.22) raised with the addition of serum HSP72 and zonulin (AUC = 0.98, *p* < 0.01; AUC = 0.79, *p* < 0.01). In a full model comprising age, sex, serum HSP72, and zonulin, the accuracy of evaluating the occurrence of CI showed significant differences between CI and healthy individuals (AUC = 0.98, *p* < 0.01). **(B,C)** Scatter diagram manifested positive correlations between NIHSS scores and serum HSP72 **(B)** and zonulin **(C)**. **(D)** Scatter diagram showed correlations between serum HSP72 and zonulin. ROC = receiving operating characteristic. AUC = area under the curve.

According to binary logistic analysis, ROC curve analysis was performed to predict CI occurrence. Strikingly, the AUC, which represented the prediction precision, raised from 0.57 in the basic model (age + sex, *p* = 0.22) to 0.98 in a way that added serum HSP72 levels, which discriminated clearly between the CI and healthy groups in the logistic regression analysis (*p* < 0.01). The AUC was 0.79 with the addition of the serum level of zonulin (*p* < 0.01), which also discriminated markedly between the CI and healthy groups in the logistic regression analysis. Furthermore, when bringing age, sex, serum HSP72, and zonulin into the full model, the AUC reached 0.98 (*p* < 0.01) (Figure 2A; Table 2).

**Table 2:** Receiver operating characteristic association statistics for forecasting cerebral infarction.

	AUC (95% CI)	*p*-value
Basic (age, sex)	0.57 (0.46–0.69)	0.22
Basic + serum HSP72	0.98 (0.96–1.00)	< 0.01**
Basic + zonulin	0.79 (0.70–0.88)	< 0.01**
Full model (age, sex, serum HSP72 and zonulin)	0.98 (0.95–1.00)	< 0.01**

AUC = area under the receiver operating characteristic curve; CI = confidence interval. ^**^p < 0.01.

### Association between serum HSP72 and zonulin levels and the link to CI severity

Based on Pearson correlation analysis, we further observed that serum HSP72 (*ρ* = 0.93, *p* < 0.01) and zonulin (*ρ* = 0.97, *p* < 0.01) positively correlated with NIHSS scores (Figures 2B,C). Of note, we found a positive correlation between the levels of serum HSP72 and zonulin within the CI group (*ρ* = 0.87, *p* < 0.01) (Figure 2D). Similarly, after adjustment for age, sex, smoking history, previous related diseases, and routine blood test results in bias correlation analysis, serum HSP72 (*ρ* = 0.93, *p* < 0.01) and zonulin (*ρ* = 0.97, *p* < 0.01) levels continuously correlated positively with NIHSS scores in patients with CI. With the multivariate linear regression model, we found that serum HSP72 (*b* = 0.06, *t* = 14.37, *p* < 0.01) and zonulin (*b* = 0.02, *t* = 7.16, *p* < 0.01) were persistently positively correlated with NIHSS scores. These data demonstrated that higher levels of serum HSP72 and zonulin were associated with a more severe degree of CI.

### Intestinal flora correlated with serum HSP72 and zonulin

**Figure 3:**
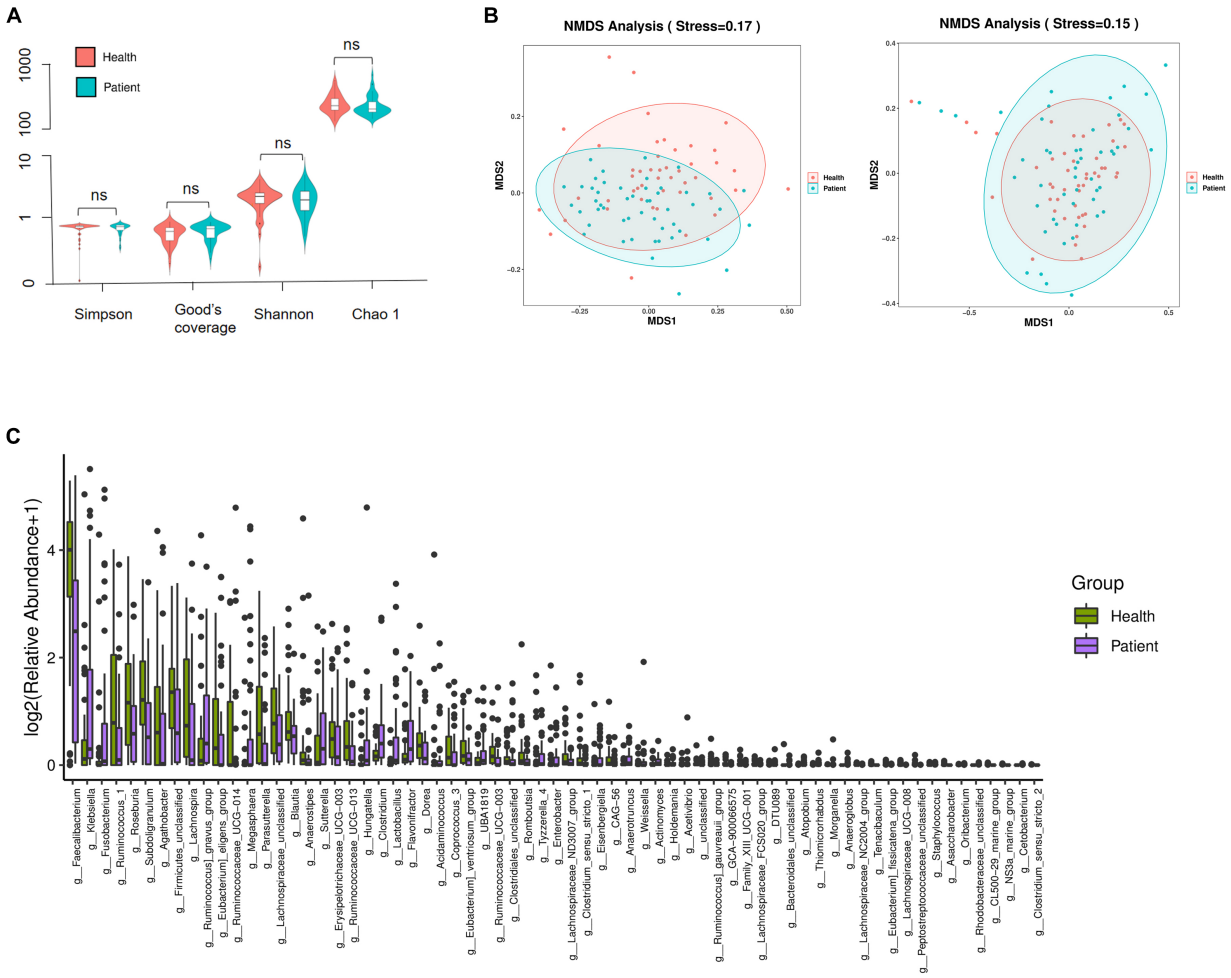
Composition and distribution of the gut microbiome. **(A)** Species richness comparison between CI and healthy groups was depicted through Chao 1, Good’s coverage, Simpson, and Shannon indexes. ns, not significant. **(B)** The differences in flora composition between samples were illustrated by NMDS. Left, unweighted UniFrac; Right, weighted UniFrac. Stress <0.2, the graphics were statistically significant. **(C)** Significance differences of 67 features at the genus level. Data were verified by the Wilcoxon rank sum test (Mann–Whitney U test).

α diversity was used to describe the species diversity of individual samples. The Wilcoxon rank-sum test, depicted through Chao 1, Good’s coverage, Simpson, and Shannon indexes, showed no statistical difference in species richness and evenness between the two groups (Figure 3A). β diversity is an index reflecting the difference in composition and distribution of bacteria between groups. NMDS plots showed significant differences in intestinal microbiota composition between the two groups (unweighted Unifrac stress = 0.17 and weighted UniFrac stress = 0.15) (Figure 3B). We then analyzed the differences in colony abundance at the 67 genus level (Figure 3C). Accordingly, the CI group was more abundant with 27 features, while the healthy group was enriched with 40 features. These data indicated that the microbial abundance in the CI group was much lower than that in the healthy group.

**Figure 4:**
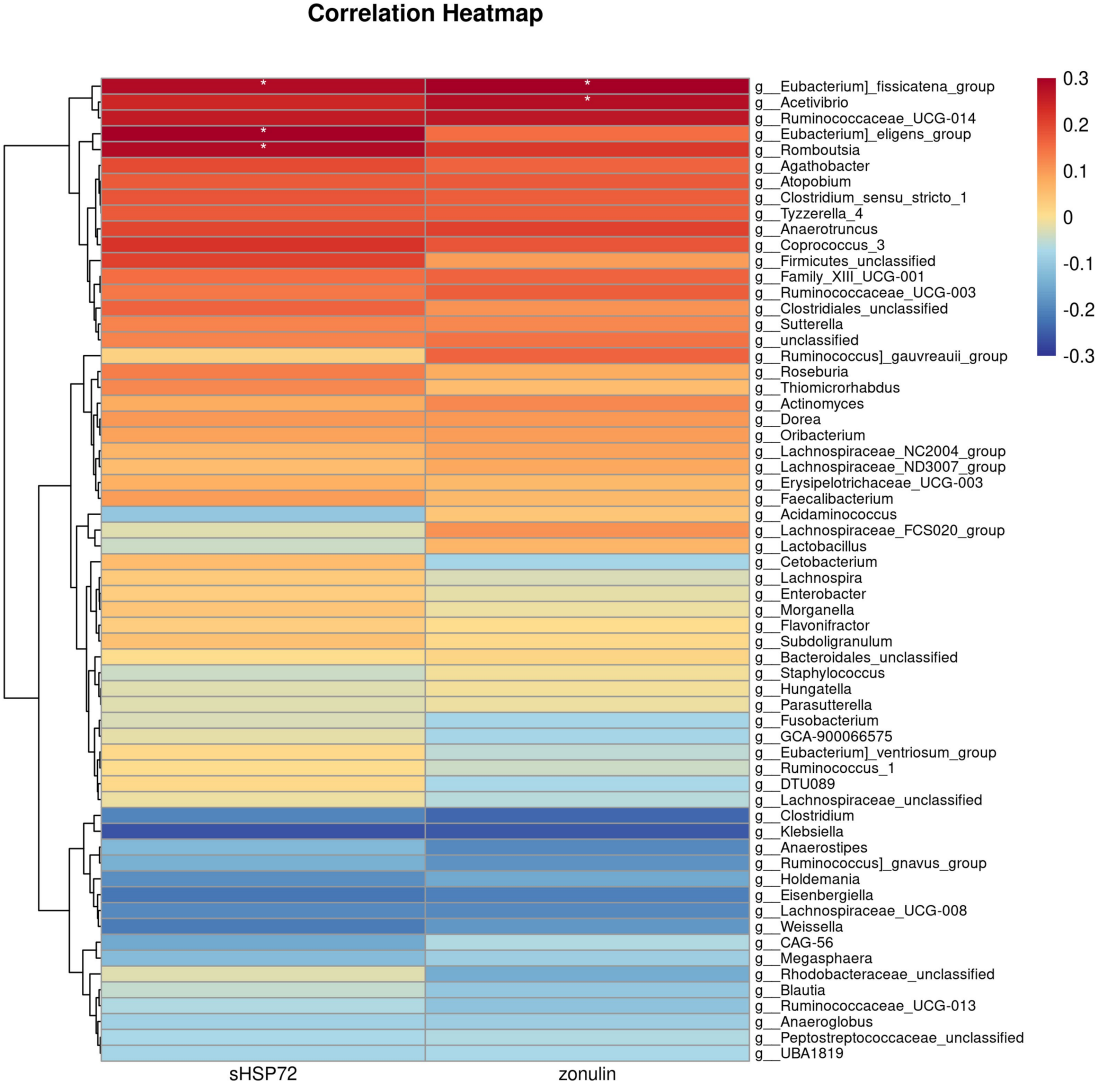
Correlation heatmap illustrating differentially abundant bacteria genus with serum HSP72 and zonulin in patients with CI. The deeper color in the figure represents a stronger correlation. The icon on the right displays the Spearman correlation coefficient gradient. Red, positive correlation. Blue, negative correlation. *, *p* < 0.05, *ρ* ≥ 0.28.

We further performed the Spearman correlation analysis on the differentially abundant bacteria genus with serum HSP72 and zonulin. We found significant correlations between the bacteria genus with both serum HSP72 and zonulin. Among the correlation heatmap for CI participants, the abundance of bacteria *Eubacterium_fissicatena_group*, *Eubacterium_eligens_group*, and *Romboutsia* manifested a remarkably positive correlation with serum HSP72 (*ρ* = 0.28, *p* = 0.04; *ρ* = 0.30, *p* = 0.03; ρ = 0.28, *p* = 0.04). The abundance of bacteria *Eubacterium_fissicatena_group* and *Acetivibrio* had a significantly positive correlation with zonulin levels (*ρ* = 0.34, *p* = 0.02; *ρ* = 0.28, *p* = 0.04) (Figure 4). Of note, the CI group was characterized by a decreased abundance of *Eubacterium_fissicatena_group*, *Eubacterium_eligens_group*, and *Romboutsia* and an increased abundance of *Acetivibrio* (*p* < 0.05).

## Discussion

The present study is the first to elucidate how both serum HSP72 and zonulin levels interrelate to alterations in intestinal microbiota and CI symptom severity in patients compared with healthy controls. After adjustment for confounders, we subsequently demonstrated that an increase in serum HSP72 and zonulin was associated with the symptom severity of CI and gut microbiota alterations.

Accordingly, we demonstrated higher levels of serum HSP72 in patients with CI than in healthy controls. Notably, the levels of serum HSP72 were positively associated with CI clinical severity. Intra-cellular HSP72 distributes in nearly every cell of the body and provides multiple cell survival functions such as restricting protein aggregation, promoting protein refolding, and acting as protein chaperoning. Current research pays attention to confirming intra-cellular HSP72 expression levels in various diseases. In this regard, serum HSP72 may confer an immunostimulatory effect that, on the one hand, facilitates innate immune responses against acute pathogenic substances, whereas, on the other hand, serum HSP72 accelerates the inflammatory process in people with various diseases such as hypertension and atherosclerosis (17). As a danger signal, circulation HSP72 is capable of specifically stimulating NO, TNF-α, IL-10, and IL-6 secretion from macrophages and neutrophils (18). It is noteworthy that HSP70 interacts with microglia and macrophages through toll-like receptors (TLRs) (17). The combination of HSP70 and TLRs induces the expression of genes encoding inflammation-associated molecules and cytokines through the activation of transcription factors activator protein 1 (AP-1) and nuclear factor κB (NF-κB) (19). In this connection, theoretically, HSP72 levels are related to the severity of inflammation and stress in organisms, which explains the higher levels of serum HSP72 in patients with severe CI. Previous research confirmed that overexpressing HSP72 facilitated a satisfactory prognosis of cerebral ischemia–reperfusion injury, which may be attributed to c-Jun N-terminal kinase 3 signaling pathway inhibition and Akt1 activation (20). George et al. found an association between HSP72 overexpression and reduced reactive astrocytes after stroke, which may contribute to neuroprotection (21). Moreover, Shailaja et al. suggested that HSP72 knockdown significantly upregulates apoptosis-inducing factors and ROS levels in both anoxia/reoxygenation (22). Recent work by Xu et al. (8) demonstrated that the upregulation of HSP72 in ischemic cerebral tissue has a crucial protective role in the outcome of stroke. In addition, it is noteworthy that endogenous HSP72 is released through necrotic or lytic cell death and activation of the α-adrenergic receptor pathway in response to environmental stressors in ways that can be detected in blood and excreta (23). We speculated that the elevation of the serum HSP72 level after CI mainly resulted from two leading causes. The first is that intracranial ischemic death cells rupture and release HSP72 to peripheral blood circulation. The other is that the sympathetic adrenal axis activated after CI triggers a large amount of HSP72 released from tissue cells and intracranial inflammatory cells to alleviate brain inflammation and oxidative damage. Recent evidence revealed that high levels of serum HSP72 were usually associated with enhanced oxidative profiles and ascending rates of mortality among septic patients (24). To this point, our results suggested that HSP72 may play a role in the deterioration of CI.

We observed that patients with CI had higher serum concentrations of zonulin than controls. Zonulin levels correlated with the degree of neurological deficit after CI. Our findings support previous studies that indicated that blood levels of zonulin are elevated in neuroinflammatory diseases, such as stroke, severe traumatic brain injury, neurodegenerative diseases, and psychiatric disorders (25). Produced by small intestine epithelium, the blood zonulin level becomes a reliable indicator reflecting intestinal permeability and inflammatory response level *in vivo*. Intriguingly, it is worth mentioning that intestinal epithelium plays an essential role in triggering the pathogenesis of numerous inflammatory diseases, and zonulin levels are provoked under acute stress states. Apart from the direct neural pathway connecting the intestine and the brain, it has long been appreciated that post-stroke dysbiosis gives rise to a down-modulated biosynthesis of γδT lymphocytes that directly impairs the immune system stability. It is worth mentioning that gut microbiota dysbiosis not only induces intestinal homeostasis damage but also stimulates the migration of γδT lymphocytes from the intestinal tract to the brain (26). Bacterial products and cellular components derived from the intestinal flora have an impact on the prognosis of patients with CI. Lipopolysaccharide (LPS) released by gut microbes after CI binds to TLRs and then activates the TLR4/P13K/Akt/MAPKs pathway, which sets the stage for matrix metalloproteinase 9 (MMP-9) expression in astrocytes and astrocytes migration and leads to intestinal leakage (27). Trimethylamine oxide (TMAO), a component of microbiota metabolite, is positively correlated with infarct size and severity of CI (28). Inflammatory signaling pathways mediated by TMAO involved NF-κB, pyrin dome-containing protein 3 (NLRP3) inflammasome, and the MAPK/JNK pathway in the peripheral and central nervous systems (29). Alternatively, the release of zonulin triggered by intestinal flora imbalance induces antigen influx from the intestinal lumen to the lamina propria and further exacerbates immune response, causing IFN-γ and TNF-α release (4, 30). Additionally, zonulin secretion was regarded as MyD88-dependent followed by protein ZO-1 dissociation from the tight junctional complex, which was responsible for both intestinal and extraintestinal inflammation, autoimmunity, and cancer (5, 6). Accordingly, the alterations of zonulin may be attributed to post-stroke dysbiosis and neuro-humoral mechanisms.

Moreover, we further observed the positive correlation between the levels of serum HSP72 and zonulin. A compromise of the intestinal mucosa is a result of increased severity and duration of stress and insufficient endogenous protective factors. Previous research has given insight into the endogenous protective mechanism of HSP72 within enterocytes. In 1999, an *in vitro* cell study conducted by Musch et al. (31) supported that HSP72 played a pivotal role in the integrity of the actin cytoskeleton and maintenance of epithelial barrier function under oxidant-induced stress. It remains a mystery whether serum HSP72 could be a reliable indicator reflecting intestinal permeability. We speculated that serum HSP72 may act on the brain-gut axis accelerating intestinal barrier destruction.

In the present study, the 16 s rRNA sequencing results demonstrated that CI was associated with certain transformations in fecal bacteria. Consistent with the findings of previous publications, post-stroke dysbiosis was characterized by reduced diversity, decreased abundance of protective bacteria, and harmful bacterial overgrowth. Intestinal dysbiosis is effectively linked to several risk factors for stroke, such as diabetes, hypertension, and atherosclerosis, and also to stroke outcomes. However, previous studies on CI and specific alterations of intestinal flora failed to obtain unanimous and definite conclusions, which may result from different patient races, different DNA detection methods used by researchers, and different patient dietary habits.

Furthermore, we discovered that the levels of serum HSP72 and zonulin in patients with CI were correlated with the relative abundance of specific differential microbial genera. The abundance of bacteria *Eubacterium_fissicatena_group*, *Eubacterium_eligens_group,* and *Romboutsia* manifested a remarkably positive correlation with serum HSP72. The abundance of bacteria *Eubacterium_fissicatena_group* and *Acetivibrio* had a significantly positive correlation with zonulin levels. Indeed, we noted the CI group was associated with a significant decrease in the abundance of *E. fissicatena group* and *E. eligens group*. The genus *Eubacterium*, belonging to the bacterial phylum *Firmicutes*, has been identified to contribute to massive aspects of human health, for the majority of the family produce short-chain fatty acids (SCFAs), especially butyric acid. It is acknowledged and accepted that SCFAs act as a special nutrient and energy component of the intestinal epithelium, protect the intestinal mucosal barrier, and reduce inflammation levels in the body. Of note, *Eubacterium* has been shown to detoxify toxic compounds into more benign forms in the intestine. Understandably, it has been reported recently that the reduction or absence of *Eubacterium* is associated with many diseases, such as depression, obesity, inflammatory bowel disease, type 2 diabetes, cardiovascular disease, and autism (32). However, the functional annotation of *E. fissicatena group* and *E. eligens group* remains poorly understood, partly because both of the bacterial species are rarely detected in feces in previous studies. Despite the protective nature of most members of the genus *Eubacterium*, recent evidence revealed that *E. fissicatena group* belongs to potentially disease-related bacteria that add to the risk of intestinal inflammation and metabolic disorders (33). Jing et al. reported that the increased abundance of *E. fissicatena group* had a positive correlation with serum TMAO levels, which was one of the independent risk factors of acute coronary syndrome (34). Nevertheless, *E. fissicatena group* was also regarded as butyrate-producing bacteria and beneficial bacteria suppressing intestinal inflammation (35). Another associated genus *E. eligens group* has been widely acknowledged to exhibit its probiotic effects. Using metagenomic analysis to estimate the gut microbiome profile in atherosclerosis patients, Sheng et al. revealed that the abundance of *E. eligens group* was positively correlated with propionate and butyrate production but was negatively correlated with inflammatory marker high-sensitivity C-reactive protein and visceral fat area. Similarly, *E. eligens group* played vital roles in the pathway CDP-diacylglycerol biosynthesis and was also significantly correlated with higher high-density lipoprotein-cholesterol levels, which significantly modulate the lipid metabolism (36). Afterward, *in vitro* cell-based assays found that *E. eligens group* efficiently promoted the production of the anti-inflammatory cytokine IL-10, suggesting the potential to be a therapeutic target for inflammatory diseases (37). In general, it seems reasonable in our results that *E. fissicatena* and *E. eligens group*, combined with serum HSP72 and zonulin, have the potential to be involved in the post-stroke systemic inflammatory response.

Our research also added to previous reports that the CI group has an increased abundance of genus *Acetivibrio*, which manifested a positive correlation with zonulin levels. The genus *Acetivibrio* was equipped with efficient biological machinery transferring lignocellulose into ethanol and has been known to ferment carbohydrates to produce acetic acid (38). Yuan et al. (39) provided evidence that *A. ethanolgignens group* played a pivotal role in facilitating inflammation and lipid metabolism abnormalities as well as interfering with the energy supply process of the tricarboxylic acid cycle. Normal peristalsis, digestive, and absorption functions of the gut require a series of coordinated operations of intestinal cells. Intestinal flora disorders and systemic inflammation will allow for intestinal barrier disruption and the invasion of harmful substances into circulation. We speculated that *Acetivibrio* accelerated increased intestinal permeability through the induction of metabolic disturbance and energy intake difficulty of intestinal cells.

Genus *Romboutsia* are SCFA producers and immunomodulators in the gut, which act in the maintenance of intestinal barrier integrity. Our results revealed that a significantly increased abundance of *Romboutsia* genus was observed in the healthy group as compared to the CI group, and the higher the abundance of *Romboutsia* genus, the higher the levels of serum HSP72. In earlier studies, Gerritsen et al. (40) showed that, as a dominant taxon in the small intestine of rats, *Romboutsia* displayed a restricted capacity to synthesize amino acids and vitamins, whereas it was adept at the utilization of different relatively simple carbohydrates (40). Intriguingly, *Romboutsia* has the potential to engage in obesity-related metabolic abnormalities. Previous studies conducted by Zeng et al. (41) indicated that *Romboutsia* was positively associated with body weight, serum lipids, and UA. We, therefore, speculated that the decreased abundance of *Romboutsia* could be an indicator of post-stroke dysbiosis and that the genus *Romboutsia* may have something to do with serum HSP72 levels and post-stroke immunomodulatory effects.

Taken together, our findings proposed that an increase in serum HSP72 and zonulin was observed in patients with CI. It has to be emphasized that the levels of serum HSP72 and zonulin were related to the clinical severity of CI and specific gut microbiota alterations. Our present study has some limitations. First, it was a single-center cross-sectional study with inevitable time and place biases. Second, considering timely bowel movements, the NIHSS scores of CI patients enrolled in this study did not reach more than 15, which made it impossible to assess the relationship between extremely severe CI and the levels of serum HSP72 and zonulin. Finally, a considerable part of patients in the CI group were accompanied by different coexisting diseases that may have affected the results. In general, our results provided promising research prospects that the levels of serum HSP72 and zonulin have the potential to serve as prospective markers for distinguishing patients with CI from controls and mirroring disease severity. Further investigation is required to explore the definitive mechanisms of how serum HSP72 and zonulin act on the process of post-stroke systemic inflammation and intestinal dysbiosis.

## Data availability statement

The original contributions presented in the study are included in the article/supplementary material, further inquiries can be directed to the corresponding authors.

## Ethics statement

The studies involving humans were approved by the studies involving human participants were reviewed and approved by the Affiliated Hangzhou First People’s Hospital, Zhejiang University School of Medicine. The patients/participants provided their written informed consent to participate in this study. The studies were conducted in accordance with the local legislation and institutional requirements. The human samples used in this study were acquired from a by-product of routine care or industry. Written informed consent for participation was not required from the participants or the participants' legal guardians/next of kin in accordance with the national legislation and institutional requirements.

## Author contributions

JZ: Conceptualization, Data curation, Formal analysis, Funding acquisition, Investigation, Methodology, Project administration, Resources, Writing – original draft. YD: Writing – original draft. BT: Project administration, Resources, Software, Supervision, Validation, Visualization, Writing – review & editing. HZ: Project administration, Resources, Software, Supervision, Validation, Visualization, Writing – review & editing.
